# Integration of human cell lines gene expression and chemical properties of drugs for Drug Induced Liver Injury prediction

**DOI:** 10.1186/s13062-020-00286-z

**Published:** 2021-01-09

**Authors:** Wojciech Lesiński, Krzysztof Mnich, Agnieszka Kitlas Golińska, Witold R. Rudnicki

**Affiliations:** 1 Institute of Computer Science, University of Białystok, Ciołkowskiego 1M, Białystok, Poland; 2 Computational Center, University of Białystok, Ciołkowskiego 1M, Białystok, Poland

**Keywords:** Machine learning, Random forest, Data integration

## Abstract

**Motivation:**

Drug-induced liver injury (DILI) is one of the primary problems in drug development. Early prediction of DILI can bring a significant reduction in the cost of clinical trials. In this work we examined whether occurrence of DILI can be predicted using gene expression profile in cancer cell lines and chemical properties of drugs.

**Methods:**

We used gene expression profiles from 13 human cell lines, as well as molecular properties of drugs to build Machine Learning models of DILI. To this end, we have used a robust cross-validated protocol based on feature selection and Random Forest algorithm. In this protocol we first identify the most informative variables and then use them to build predictive models. The models are first built using data from single cell lines, and chemical properties. Then they are integrated using Super Learner method with several underlying methods for integration. The entire modelling process is performed using nested cross-validation.

**Results:**

We have obtained weakly predictive ML models when using either molecular descriptors, or some individual cell lines (AUC ∈(0.55−0.61)). Models obtained with the Super Learner approach have a significantly improved accuracy (AUC=0.73), which allows to divide substances in two categories: low-risk and high-risk.

**Supplementary Information:**

The online version contains supplementary material available at (10.1186/s13062-020-00286-z).

## Background

Drug-induced liver toxicity is a common cause of liver injury. It accounts for approximately half of the cases of acute liver failure. What is more, it mimics all forms of acute and chronic liver disease. DILI often presents as acute hepatitis and/or cholestasis; nevertheless, virtually any clinicalpathological pattern of acute or chronic liver disease can occur. Each drug associated with hepatotoxicity tends to have a characteristic signature regarding latency and pattern of injury [1].

The mechanism can be arise due to drug metabolism or it can be related to the chemical properties of the drug molecule itself [2]. Kamplowitz et al. estimated that over one thousand drugs have been implicated in causing liver disease on more than one occasion [3]. DILI is a significant clinical problem in terms of patient morbidity and mortality and also represents a challenge for the pharmaceutical industry leading to attrition of drugs in development and withdrawal of drugs post-licensing [4].

In addition to placing patients in harm’s way, the economic impact of DILI to stakeholders (i.e. patients, healthcare system, regulatory agencies, pharmaceutical industry) is significant (3-5 billions of dollars, 12-15 years per successful drug) [5]. Preclinical drug studies in animals are often inadequate to evaluate human DILI because of significant species-specific differences in liver functions, such as drug metabolism pathways. Consequently, in vitro human liver models including microsomes, cell lines, primary human hepatocytes (PHHs), and liver slices are used to supplement animal testing [6].

Multiple approaches were examined for DILI prediction. Vorrik et al. [7] proposed experimental approach, using 3D spheroid cultures of primary human hepatocytes in chemically defined conditions for DILI prediction. Albrecht et al. [8] predicted DILI in relation to oral doses and blood concentrations. They created two metrics: the toxicity separation index and the toxicity estimation index and use support vector machine for classification. Other studies relied on data collected in databases and Machine Learning methods to derive predictive models. In particular Hong et al. [9] used decision forest based on FDA’s Liver Toxicity Knowledge Base for DILI prediction. Muller et al. [10] used standard Machine Learning to predict DILI, relying on in vivo models of DILI of organic molecules. Certain descriptors in this model were both measured experimentally in vitro and calculated theoretically from the molecular structure.

The DILI prediction problem was investigated in the 2018 CAMDA challenge. In this case two human cell lines: MCF7 and PC3, were tested. Chierici et al. created a deep learning architecture for DILI prediction based on MCF7 and PC3 human cell lines [11]. The authors obtained results slightly better than random ones - MCC equal 0.19 in the best case. In work [12] the same problem was solved by 7 various classifiers. Prediction results were similar to the previous one, with accuracy = 0.7 and MCC = 0.20. Both works mentioned above performed binary DILI classification based on 3 classes DILI division (most DILI concern, less DILI concern, no DILI concern).

Current study was performed within the framework of the CAMDA 2019 CMap Drug Safety Challenge. The toxicity of drugs was specified by classification from Federal Drug Administration (FDA) [13]. It is derived from analysis of the hepatotoxicity descriptions presented in the FDA-approved drug labeling documents, and from assessing causality evidences in literature. This dataset is the largest publicly available database of DILI annotations. Drugs with confirmed causal evidence linking a drug to liver injury are classified into three groups (Most-, Less- and No-DILI concern). Additionally, drugs for which the definitive causal link is undetermined are labelled as Ambiguous-DILI-concern. The CAMDA toxicogenomics challenge aimed at creating predictive models for DILI that would provide estimates of risk of DILI for new compounds. The training data consisted of gene expression profiles for several cell lines exposed to drug compounds. Additionally, the molecular structures of drug compounds were provided. In the current study we addressed the challenge by proposing a robust protocol for deriving robust machine learning models of DILI.

## Materials and methods

### Data

The DILI classification is provided for 233 of these compounds, using two related classification schemes based on the mentioned above FDA classification [13]. In the first one four classes are defined:

1. *most DILI concern* – 39 compounds,
2. *less DILI concern* – 90 compounds,
3. *ambiguous DILII concern* – 50 compounds,
4. *no DILI concern* – 54 compounds.

In the second one classes 2. and 3. are merged into a single class *less DILI concern*.

Three types of descriptive data were provided for drug compounds with DILI classification:

- molecular structures (1660 variables based on SMILES code [14],
- gene expression profiles for thirteen cell lines exposed to these compounds (12328 genes for each human cell line),
- and annotated images from cellular assays [15, 16] for a subset of drug/cell lines combinations.

Unfortunately, the subset of compounds with known decision and image assays was limited to 156, and therefore we decided to omit this data in the analysis, concentrating only on the data that was available for the full set of compounds. Gene expression data sets and molecular structures were provided for 233 drug compounds, 179 with non-zero DILI concern level and 53 with no DILI concern.

The human cell lines treated by drug compounds, which were provided to challenge participants, are listed in the Table 1.

**Table 1:** Cell lines used in the current study. All cell lines with exception of PHH are derived from cancer cells. PHH, on the other hand, is considered to be the gold standard for hepatic in vitro culture models

Symbol	Description	Symbol	Description
A375	human melanoma	ASC	adipose stromal cell
HA1E	human embryonic kidney	HCC515	lung cancer
HPEG2	human liver cancer	HT29	human colon cancer
MCF7	breast cancer	NPC	vasopharyngeal carcinoma
PC3	human prostate cancer	SKB	human breast cancer
VCAP	human prostate cancer	PHH	primary human hepatocytes
A549	adenocarcinomic human alveolar		
	basal epithelial cells		

The experimental protocol for measurement of gene expressions was not uniform: the cell lines were exposed to various doses of examined compounds, and measurements were performed at three different incubation times after exposure (6, 24 and 48 h). Unfortunately, the number of measurements varied strongly between compounds and cell lines, i.e. for any given combination of compound and cell line there can be one measurement taken with a single dose, or three measurements for several doses. Since information on what dose and which incubation time are appropriate for each drug, a simple unifying approach was applied. Only one measurement for each compound was taken into account. It was always a measurement taken with the highest available dose. The most commonly used incubation time of 24 h was used, unless it was not available. In such a case, we used 6 h incubation and if not available then 48 h incubation was used. This approach was based on simple assumption, that all biological effects that can be related to DILI should be more intensive for larger doses of compound. The 24-hours incubation was selected, because it was by far the most common, followed by 6- and 48-hours incubation. The protocol for selection of samples described above resulted in most uniform data set possible within the data provided for the challenge.

Gene expression for the study was generated using L1000 Platform [17], developed for Connectivity Map [18] at the Broad Institute. The Connectivity Map (also known as CMap) is a collection of genome-wide transcriptional expression data from cultured human cells treated with bioactive small molecules. It was developed to enable the discovery of functional connections between drugs, genes and diseases through the transitory feature of common gene-expression changes. L1000 is a gene-expression profiling assay based on the direct measurement of 978 genes that constitute a reduced representation of the transcriptome. Then nearly twelve thousand additional gene expression profiles are inferred computationally. The number of landmark transcripts whose abundance is measured directly is approximately one thousand. Eighty additional invariant transcripts are also explicitly measured to enable quality control, scaling and normalization. Measurements of transcript abundance are made with a combination of a coupled ligase detection and polymerase chain reaction, optically-addressed microspheres, and a flow-cytometric detection system.

Drug compounds tested for Drug Induced Liver Injury were described by SMILES (Simplified Molecular-Input Line-Entry System) [14]. To derive their chemical predictors we used molecular descriptor calculator Mordred ver. 1.1.1 [19], provided within Python environment. Mordred computed 1660 physical and chemical molecular descriptors using both 2D and 3D representations of molecules.

### Modelling procedure

**Data Integration** The current study uses two approaches to data integration. First, we separately build individual models for each data set. Then, we apply early integration strategy [20] to combine each gene expression data set with molecular descriptors to obtain heterogeneous models. Finally, we use late data integration strategy [20] for combining these heterogeneous models using gene expression from different cell lines into a single final model with the help of super learning methodology [21].

**Repeated cross validation and nested cross validation were used to obtain unbiased estimates of performance and variance of modelling approaches.** Machine learning methods very often produce models that are biased towards training set. In particular, selection of hyper-parameters of the algorithms and selection of variables that will be used for modelling can introduce strong biases. What is more - a simple selection of best performing model also can lead to a bias. Finally, the simple act of dividing data set into training set and validation set involves bias by creating two partitions with negative correlations between fluctuations from the true averages [22]. To minimize influence of biases, and estimate variance of the models the following process of model building was performed within multiple repeats of cross-validation loop:

- split the data into training and validation set;
- identify informative variables in the training set;
- build model on the training set using most informative variables;
- estimate quality of the models on the validation set.

In the final step of modelling, prediction results based on particular cell lines were combined into a single prediction, using the super learning methodology proposed by van der Laan et al. [21]. Super learning utilises results of an internal cross-validation for individual learning algorithms and merges them into a single prediction. To verify these predictions we used nested cross validation, i.e. the entire super learning protocol was run on the training sets of the external cross-validation and tested on its validation sets. This allowed us to obtain unbiased estimates of performance and variance of super learning results. See the last paragraph of this section for more details about the super learning protocol.

**The identification of informative variables was performed with the help of two methods:** Welch t-test for differences in sample means [23], or multidimensional filter based on information theory developed in our laboratory [24, 25] and implemented in the R package *MDFS*. MDFS allows to identify variables involved in non-linear and multidimensional interactions with the decision variable. Two variants of MDFS were used: one-dimensional (MDFS-1D) and two-dimensional (MDFS-2D). In particular, MDFS-1D can identify variables that interact with decision variable in non-linear fashion, whereas MDFS-2D allows to identify the variables that gain importance due to interactions with other variables. To avoid false positive results, all filters apply corrections using the number of variables in the data set as the parameter describing number of independent tests. In the presence of numerous correlated variables the number of independent tests is smaller and hence the apparent number of relevant variables may be too low or in extreme cases filters may not return relevant variables at all. For consistency of procedure, we simply used 100 highest-scoring variables are used to build predictive models. In our experience, this approach gives reasonable results and does not lead to overfitting when there is no true information in the system. The comparison of classification results for the data sets comprising of gene expression profiles from individual cell lines as well as the molecular descriptors showed that the best results were obtained using a one-dimensional variant of MDFS for gene expression, and t-test for molecular descriptors. Hence, these feature selection algorithms were used for the appropriate types of data for the later stages of modelling.

**Models were obtained with the help of Random Forest (RF) classification algorithm [**26]. It is a classifier that works well *out of the box* on most data sets [27] It is relatively robust when the number of variables is very large. Nevertheless, in our experience, RF models are better and more robust on gene expression data, when only informative variables are used. In particular, both model quality, and computational performance are degraded when a very large number of variables is used. Best results are usually obtained, when the number of variables is limited to 100. Both types of data contain a huge number of descriptive variables - 12328 in the case of gene expression profiles, and 1660 molecular descriptors. Hence, it is important to limit the data to contain only informative variables.

**Two performance measures, namely area under receiver-operator curve (AUROC, AUC) and Matthews Correlation Coefficient (MCC) were used to assessing quality of machine learning models.** The MCC belongs to the group of balanced indicators of accuracy, such as F1 score or balanced accuracy, that take into account number of good predictions in both classes. It is the only measure that properly takes into account the relative sizes of the classes [28]. The AUC is a global measure of performance that can be applied for any classifier that ranks the binary prediction for objects. Both measures are symmetrical – their value depends on performance of classifiers for both classes. It has been argued that AUC is less suitable than area under Precision-Recall curve (AUPRC) [28**–**30] for computational biology and medicine applications. However, the relative advantage of AUPRC pertains to cases where either: there is huge imbalance between rare interesting cases among of deluge of non-interesting ones, or the costs/benefits are hugely disproportional for false positive and false negative cases (for example in testing for cancer). One can argue that costs and benefits for DILI are disproportional – false negatives (failing to recognise DILI causing drugs) are causing visible harm, whereas predicting high DILI potential for a harmless molecule does not produce any visible harm. This indeed may be the case for drugs used for treatment of mild diseases, where harm due to DILI may be much larger than any benefits from using the drug in question. However, the calculus of harms and benefits may be reversed for potentially life-saving drugs that may also lead to mild DILI, in particular, when there is no alternative therapy. For each and every drug and disease pair, the balance between harms and benefits is different. Hence, the selection of quality measures that are symmetrical with respect to positive and negative cases is appropriate.

**The prediction results based on particular cell lines were combined into a single prediction, using the super learning methodology proposed by van der Laan et al. [**21].

The super learning method uses an internal cross-validation to compute unbiased predictions of probability, that a substance is harmful for the liver, based on the individual data sets. The predictions are then treated as new explanatory variables and used to build the second-order machine learning model. To obtain the eventual results for new data, one should first compute the individual predictions, then apply the second-order model for them.

The combining model was built using the following methods:

- choice of the best-performing single classifier;
- mean of all the results;
- mean of 5 best results;
- linear combination, based on Linear Discriminant Analysis principle [31], with non-negative weights;
- applying Random Forest machine learning algorithm.

To improve the stability of the results, we applied repeated cross validation in super learning. The choice of the single best-performing classifier and the 5 top-rated models was based on the average values of area under ROC curve (AUC) over 5 repeats of 10-fold cross validation with diverse splits. The weights of the linear model and the final results of the Random Forest classification were averaged over repeats of cross validation.

**Fig. 1:**
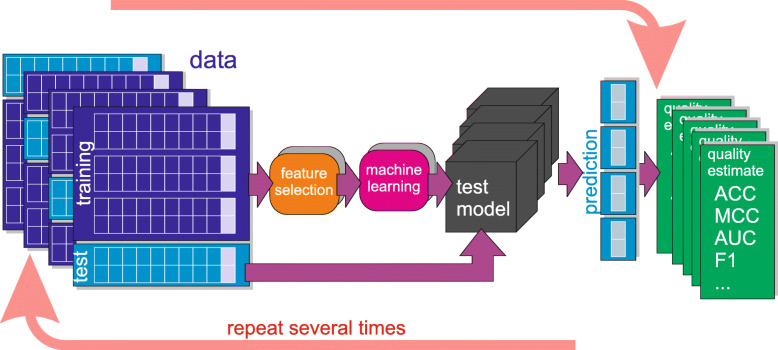
Nested cross validation scheme

The performance of the combined classifiers was estimated, using the nested cross validation protocol. The entire modelling routine, including feature selection, computing cross validated predictions based on single cell lines and building the ensemble predictive model, was performed on the training sets of the external cross validation and tested on its validation sets. We performed 20 repeats of 10-fold external cross validation. As a reference, we computed also a biased estimate of performance of the ensemble models, using the training data sets. The schematic representation of the nested cross validation procedure used in the current study is displayed in Fig. 1.

## Results and discussion

### Binary classes definition

We decided to use an aggregated classification scheme with a binary split between final classes. Several methods of aggregation were tested:

- Class 1 as *DILI concern*, classes 1, 2, and 3 as *no DILI concern*;
- Classes 1 and 2 as *DILI-concern*, classes 3 and 4 as *no DILI concern*;
- Classes 1 and 2 class as *DILI-concern*, class 4 as *no DILI concern*;
- Classes 1 and 2 as *DILI-concern*, class 4 as *no DILI concern*;
- Classes 1, 2, and 3 as *DILI-concern*, class 4 as *no DILI concern*.

The best results were obtained for the last method. In this aggregation 179 compounds are assigned to class *DILI-concern* and 54 compounds to class *no DILI concern*. Removing class 3 (*ambiguous DILI concern*) did not improve results, to the contrary, slightly worse results were obtained in comparison with the last aggregation method.

### Hyperparameters selection and feature number fixing

**Fig. 2:**
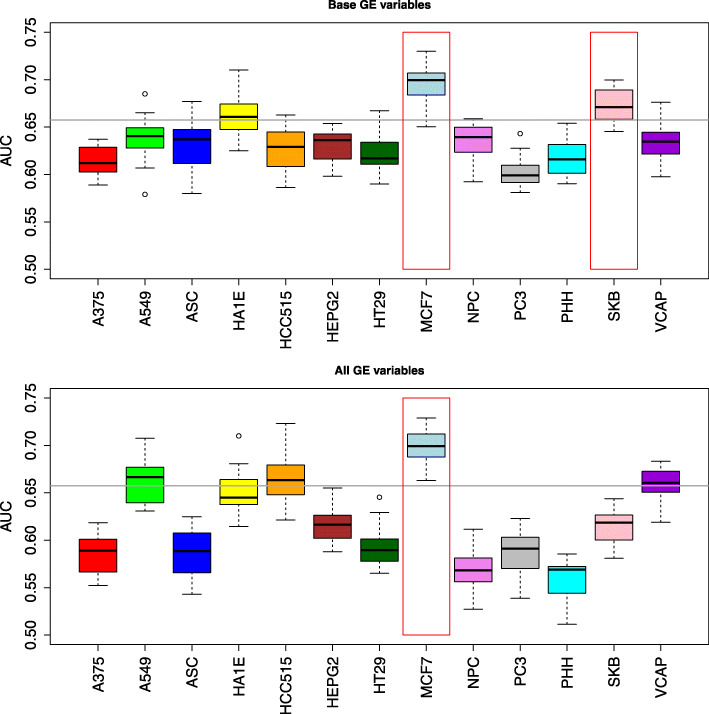
Box plots of cross validated AUC for integration of gene expression patterns for various human cell lines and chemical structures of drugs. Horizontal gray line denotes the mean AUC obtained from chemical descriptors alone. Red frames indicate cell lines, that contribute significant information to the chemical descriptors

**Fig. 3:**
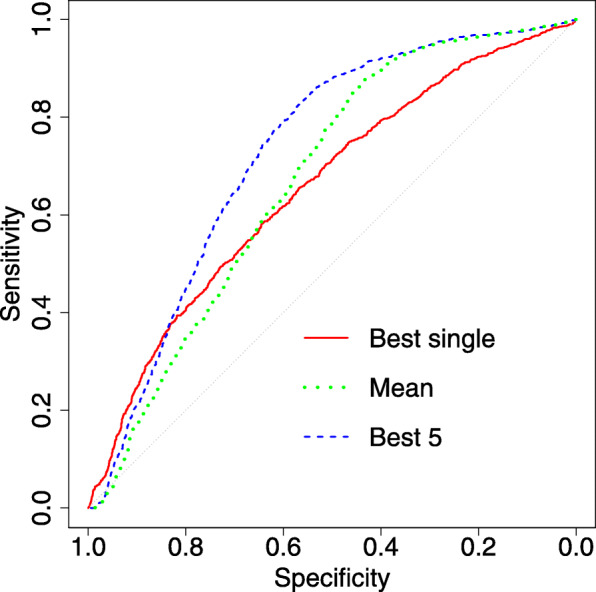
ROC plots and box plots of AUC for the best single model,mean of 13 models and mean of 5 best models in nested cross validation

**Fig. 4:**
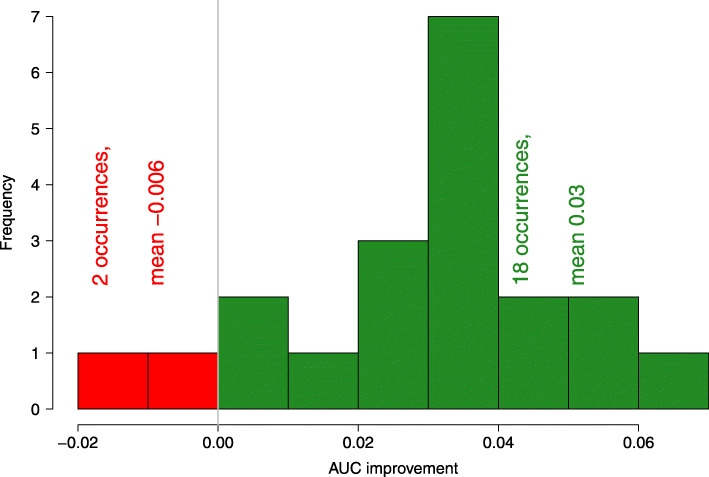
Histogram of the difference of AUC between the mean of 5 best models and the best individual model i.e. gene expression for MCF7 cell line integrated with chemical descriptors, over 20 repeats of the external cross validation

In most cases, our feature selection methods reported no relevant variables in gene expression data sets. Nevertheless, for some cell lines, the number of very weakly informative variables greatly exceeded the expected values, see Figure 1 in the Additional File 1. For example, for the MCF7 cell line, the expected false discovery rate for 100 most relevant variables was near 0.5, suggesting that there are about 50 truly, albeit weakly, informative variables within the 100 most relevant ones, see Figure 2 in the Additional File 1. On the other hand, the measured relevance of variables on the PC3 cell line conformed to the theoretical distribution, and expected false discovery rate is close to 1, see Figures 3 and 4 in the Additional File 1. Therefore, all models were built using top N highly ranked descriptors, with the value of N established experimentally, see Table 1 in the Additional File 1. In the case of molecular descriptors obtained from the Mordred, the number of relevant variables obtained for the entire data set is 127 when FDR level 0.1 was applied, see Figures 5 and 6 in the Additional File 1. The number of relevant variables obtained in cross-validation varies between folds. We used top 100 variables for consistency between folds and also with gene expression data.

The value of the mtry parameter of Random Forest, corresponding to the number of variables tested at each split creation, was established experimentally outside of the cross-validation loop. All values from the (2, 20) interval were tested and AUC was used as a quality metric, see Table 2 in Additional File 1. The quality of the results was generally not dependent on the selection of mtry, except when very small values were used. Therefore, the default value of mtry was used throughout the study.

### Individual models and data integration

The initial models were built using all expression data available for the single cell line. Exposures to different concentrations and different measurement times of a single drug compound were treated as independent data points. This approach leads to significant overfitting, since responses to different doses of the same compound may be correlated, even when taken at different times. Indeed, one can observe in the Table 2, that the apparent quality of results is strongly correlated with the size of the data set, hence with the redundancy in the data.

**Table 2:** Results of prediction on entire data sets. Columns 3, 4: results obtained in a simple cross validation. Columns 5, 6: results obtained in a modified cross validation, where subsets contain either all or none of the observations for a compound

		Simple CV		Clustered CV	
Cell line	Number of observations	MCC	AUC	MCC	AUC
A375	870	0.43	0.70	0.01	0.57
A549	1335	0.46	0.70	-0.04	0.54
ASC	286	0.27	0.53	-0.04	0.48
HA1E	944	0.32	0.62	0.01	0.56
HCC515	834	0.33	0.58	-0.04	0.50
HPEG2	551	0.30	0.61	-0.01	0.54
HT29	825	0.41	0.71	-0.03	0.60
MCF7	2298	0.52	0.72	-0.06	0.54
NPC	489	0.35	0.63	-0.05	0.56
PC3	1679	0.48	0.69	-0.04	0.54
PHH	284	0.15	0.53	0.00	0.50
SKB	334	0.28	0.62	0.05	0.59
VCAP	1325	0.53	0.72	-0.01	0.57

To estimate the effect of overfitting due to the correlated observations, we repeated the procedure in a cross validation appropriate for clustered data [32]. Here, the training and validation sets contain either none or all the observation for a compound. The results for this test are much worse, and negatively correlated with the sample size. This suggests, that building machine learning models for the pooled results does not lead to credible results.

Therefore, to avoid the overfitting described above, we applied the modelling protocol described earlier to a data set, where each compound was represented by a single observation.

As can be expected, the apparent quality of models was lower in this case, see Table 3. The best results were obtained for MCF7 cell line with *AUC*=0.62 and *MCC*=0.23 measured in fully cross-validated procedure. Additionally, the results obtained for VCAP, A549, HA1E, HCC515 and SKB cell lines suggest weak but non-random association with DILI signal. Results for other cell lines were very weak and not significantly different from random.

**Table 3:** Results of prediction on non-redundant data sets obtained in standard cross validation procedure. Results for both for models using gene expression only (columns 2, 3 and 6, 7) and models built on integrated data sets (columns 4, 5 and 8, 9) are shown. Results for model built on molecular descriptors shown in the last row

		AUC				MCC		
Cell line			GE all	GE base			GE all	GE base
	GE all	GE base	+ chem	+ chem	GE all	GE base	+ chem	+ chem
A375	0.48	0.48	0.59	0.62	-0.01	0.00	0.05	0.12
A549	0.57	0.47	0.66	0.64	0.08	-0.07	0.13	0.09
ASC	0.47	0.50	0.59	0.63	-0.01	0.02	0.05	0.13
HA1E	0.58	0.59	0.65	0.66	0.10	0.12	0.17	0.19
HCC515	0.56	0.45	0.66	0.63	0.09	-0.03	0.16	0.07
HPEG2	0.51	0.53	0.62	0.63	0.02	0.05	0.09	0.09
HT29	0.48	0.49	0.59	0.62	-0.01	0.02	0.07	0.10
MCF7	0.62	0.62	0.70	0.70	0.23	0.18	0.29	0.23
NPC	0.42	0.43	0.57	0.64	-0.07	-0.07	0.02	0.09
PC3	0.43	0.44	0.59	0.60	-0.06	-0.03	0.06	0.07
PHH	0.42	0.44	0.56	0.62	-0.10	-0.08	0.03	0.07
SKB	0.50	0.51	0.61	0.67	0.03	0.14	0.08	0.17
VCAP	0.58	0.51	0.66	0.63	0.06	0.03	0.12	0.11
molecular descriptors	0.66				0.15			

#### Integration with chemical properties of drugs

For our analysis we obtained also over 1600 variables from SMILES (Simplified Molecular-Input Line-Entry System) description of drug. Models build only on chemical descriptors gave results slightly better than the best results obtained for human cell lines, see Table 3.

In the next we generated models using both gene expression and chemical properties. To this end, top 100 most informative variables from gene expression (obtained with MDFS), and top 100 molecular descriptors (obtained with t-test), were used to build the RF models. Experiment was carried using either top 100 from all 12328 genes, or top 100 from 978 base genes from L1000 assays.

Feature selection was more stable for chemical descriptors. One variable from this data set appeared in the top 100 in all cross validation folds, while 44 appeared in half or more cross validation folds. Most of them belong to topological structure descriptors and Burden matrix properties. Human cell lines gene expression gave worse results. In the case of MCF7, best among human cell lines, only 16 variables were chosen in at least 50 percent of top 100 descriptors sets.

The models were obtained in 20 repeats of ten folds cross validation procedure to allow for unbiased estimate of performance. As can be expected, results better than for models build only on gene expression data were obtained. With the exception of MCF7, VCAP, A549 and HA1E, the models obtained on the combined data sets were mostly no better than models obtained using molecular descriptors alone, see Table 3. Only for the MCF7 cell line, was the combined model statistically significantly better than the model built on molecular descriptors. The statistic for paired t-test over the repeats of cross validation was 9.7 for all the MCF7 variables, and 7.6 for base variables. For the SKB cell line, the results are ambiguous: statistically significant improvement (t=5.1) was observed for models built of base variables, while models using all the gene expression variables performed worse than those built on molecular descriptors. Poor performance of models built on SKB cells alone suggests that the result for base variables should be treated as an outlier, see Table 3 and Fig. 2.

Apparently presence of descriptive variables representing gene expression in most cases is not helpful and decreases the performance of Random Forest algorithm. Moreover, models using variables selected from all 12328 gene expression profiles are generally better for cell-lines for which the predictive models can be built using gene expression data alone, whereas, for other cell-lines models using variables selected from 978 base variables are better. This effect probably also arises due to cancellation of noise for non-informative cell-lines, which was more effective for base variables.

#### Straightforward integration of data from multiple cell lines

For integration of the information available in the multiple cell-lines we first tried the straightforward extension method described above. We simply built predictive model using single data set comprising 100 most relevant variables from each cell line, as well as 100 most relevant molecular descriptors. Unfortunately, this model was no better than the best model obtained for MCF7 gene expression with molecular variables. In the second iteration of straightforward integration, the only top 100 most relevant variables from the MCF7, VCAP, A549, HA1E and HCC515 cell lines, which did not decrease quality of the model based on molecular descriptors. Model created on this five cell lines achieved same quality as model built on MCF7.

#### Signal transferability

At this stage we have also examined, whether biological information obtained for one cell line is transfereable to another cell line. To this end, we performed feature selection on one cell line and used selected most informative genes to build a RF model on another cell line. These tests were carried out for all cell line - cell line combinations. Most of models built in this way gave random results (*AUC*≈0.5). Nevertheless, in few cases we obtained some informative models. Best result (*AUC*=0.58) was achieved by using variables from SKB cell line used on MCF7 cell line. Several other models built on MCF7 also are non-random. The average AUC for all pairs that did not include MCF7 is 0.50, with standard deviation 0.03.

Results for best cell line pairs are in Table 4. Additionally we examined, how similar were the rankings of relevant variables obtained for different cell lines, and in particular for these pairs of cell lines, for which non-random models were obtained. First analysis was performed for top 100 variables of both cell line pairs, but very small number of common variables were obtained, varying between 0 and 9, even for pairs with best transferability of models.

**Table 4:** Signal transferability. The number of common variables in top *N* variables of one cell line in top 200, variables is shown. The last column shows the AUC of a model built for the second cell line, using 100 most informative variables from the first cell line. The cell line used for model building, and AUC of the model are displayed in boldface

top *N* cells	top 200 cells	*N*=10	*N*=20	*N*=50	*N*=100	*N*=200	AUC
VCAP	**MCF7**	0	2	6	19	40	**0.58**
MCF7	**VCAP**	4	6	9	19	40	**0.56**
SKB	**MCF7**	1	1	7	14	34	**0.58**
MCF7	**SKB**	1	4	15	21	34	<**0.56**
HA1E	**MCF7**	2	4	10	22	40	**0.57**
MCF7	**HA1E**	2	6	12	19	40	<**0.56**
HEPG2	**MCF7**	3	4	10	18	30	**0.57**
MCF7	**HEPG2**	0	2	9	13	30	<**0.56**

Therefore, we examined how many common variables is in top *N* variables for any given cell line in top 200 variables in all other cell lines, see Table 4. In this way we account for correlations between variables — the variable that belongs to top 100 most informative variables in cell A, may still carry information for cell B, even if it does not belong to top 100 most informative ones. The limit 200, as well as thresholds in Table 4, are arbitrary, they were selected just to show trends in similarity not to imply true relevance. The number of common variables at threshold 100 shows how many somewhat informative variables were available for model building on the second cell line. For all pairs listed in the Table 4 there are between 14 and 27 variables in top 100 of the other cell line, that are also somewhat relevant for the MCF7 and can be utilised by classification algorithm to build non-random model.

### Super learner

The prediction results based on particular cell lines were combined into a single prediction, using the methodology proposed by van der Laan et al. [21]. This procedure includes verification of the results by cross-validation, hence entire modelling procedure described earlier had to be repeated multiple times within cross-validation loop. Therefore nested cross validated models for all cell lines were build. Among the 4 possible configurations: all gene expression, base gene expression, all gene expression plus chemical properties and base gene expression plus chemical properties, we chose the series of all gene expression integrated with chemical descriptors. This configuration seems to utilise the predictive ability of both gene expression and molecular descriptors (see Table 3).

Application of Super Learner approach to integrate various models resulted in modestly but significantly better models, than best models built using information from single cell line, see Table 5.

**Table 5:** Results for composite predictive models. The method for combination is displayed in the first column, the estimate of AUC obtained on the training set and in cross-validation in the second and third column. Columns four and five present results of comparison between the predictions of composite models and the best individual model i.e. MCF-7+molecular descriptors, by paired *t*-test

	AUC			
Method			Comparison with MCF-7+chem.	
	Internal CV	Nested CV	*t* statistic	*p*-value
Best single result	*0.69*	0.65	-6.8	1
Mean of all results	*0.66*	0.67	-7.0	1
Mean of 5 best results	*0.72*	0.73	6.8	9·10^−7^
Linear combination	*0.73*	0.70	-0.46	0.67
Random Forest	*0.84*	0.71	1.8	0.05

The best results were obtained when composite model was built as the average of five best elementary models. More sophisticated methods, namely non-negative linear regression, and Random Forest classifier, resulted in overfitted models that scored better when evaluated on the training set, but were worse when evaluated in the nested cross-validation. Interestingly, even the simple mean of all models was better than best single result (see Fig. 3). Apparently, the averaging procedure allowed to extract common information from different models, at the same time cancelling the noise. Still, results for mean of all models were worse than all other combination methods. Most likely, this particular method of combining results resulted in model that was skewed towards molecular descriptors, since they were the source of information in models using non-informative cell lines.

Another interesting observation arises from comparison of Tables 3 and 5. The best result in cross-validation was obtained for model built using variables from MCF7 gene expression integrated with molecular descriptors. The cross-validated estimate of AUC for this model is 0.70. The cross-validated estimate of *best single result* in Super Learner, is 0.65. This happens because the Super Learner *does not know* which model is best overall. It selects the best on a given training set in particular fold of the external cross-validation. It may happen, that for some training sets, some other classifier (for example one using VCAP gene expression variables) may give slightly better results due to random fluctuations. In such a case the other classifier will be used as the best and its prediction on the validation set will be measured. The fluctuations on the training set and validation set are negatively correlated by construction.

Therefore, when the training set contains cases more suitable for *the second best classifier* allowing it to surpass the *the best classifier* on the training set, then by definition it will have the less suitable cases in the validation set, what degrades the performance. This effect can be expected when predictions of classifiers are tested on the external data — the classifier that is best performing on the training set will not necessarily perform best on new data, and generally will have lower performance on external data. The average of 5 top-rated models appears to be robust to this effect, hence it proved superior even over the best individual classifier overall, see Fig. 4.

Our eventual, recommended predictive model is then an average of Random Forest predictions for 5 data subsets, each containing 100 chemical descriptors and 100 gene expression patterns from the cell lines that performed best in cross validation: MCF7, HCC515, A549, VCAP, HA1E. The values of AUC shown in Table 5 suggest, that the estimate of performance for the average of 5 top-rated predictors, based on the training data, is very close to the result of the external cross validation. This estimate of AUC for the model described above is equal to 0.74±0.04. The expected variation of AUC for new data was estimated using method proposed by Xu at al. [33]. Therefore, on new data one can expect *AUC*∈(0.66,0.82).

#### Risk classes and results enrichment

The quality of final model is certainly not sufficient for predicting DILI status of any compound with good precision. Nevertheless, use the prediction of the classifier to divide compounds into two, equally numerous, categories: higher- and lower-risk of DILI. Then we can compare the prevalence of all DILI concern classes in both categories. In particular, we can compute enrichment of DILI – concern classes in low-risk category over their with prevalence in the high-risk sample. The results of such procedure are shown in Table 6.

**Table 6:** Enrichment of DILI concern classes of compounds in low-risk category in comparison with high risk category

Method	Enrichment			
	no DILI	Ambiguous	less DILI	most DILI
Best single result	1.94	1.01	0.74	0.70
Mean of all results	2.32	0.82	0.65	0.96
Mean of 5 best results	3.95	0.77	0.59	0.78
Linear combination	2.81	0.86	0.65	0.71
Random Forest	2.93	0.82	0.69	0.70

In best case, with mean of best 5 classifiers combining, enrichment of predicted non-DILI concern is equal 3.95, while all DILI-concern classes are significantly depleted.

Other combining methods also achieved significant enrichment in separating no-DILI class. That results can be used as a indication in next steps in drug development.

## Conclusions

Weak predictive models for DILI can be obtained using either gene expression profiles of some cell lines exposed to drug compounds or molecular properties of these compounds. Five cell lines out of thirteen used in experiments are suitable for building predictive models, however, the model built using the chemical and physical properties of the compounds has better results than models built on any individual cell line. Integration of gene expression profiles obtained for a single cell line with chemical properties of drug compounds lead to small improvement of model’s quality in comparison with best individual model only for a two cell lines, namely MCF7 and SKB. Transferability between cell lines were observed constitutes an idependent proof that the weak signal observed in the gene expression is real. What is more, composite classifiers obtained by averaging results over several cell-lines are significantly better than individual models. The quality of the final models is not sufficient for effective prediction of DILI status of individual compounds, however, it can be used as additional information during drug development. There are several lines of further investigation that could improve the quality of predictions — identification of new informative cell lines, extended set of molecular descriptors, additional modes of information, including for example structural information on molecules and potential targets, metabolomic profiles etc.

## Supplementary Information

**Additional file 1** Supplementary materials for “Integration of human cell lines gene expression and chemical properties of drugs for Drug Induced Liver Injury prediction”

## Data Availability

The CMap Drug Safety Challenge data can be downloaded from the CAMDA 2019 Website: http://camda2018.bioinf.jku.at/(accessed in April 2019).

## Abbreviations

AUC: Area under receiver-operator curve
CAMDA: Critical assessment massive data analysis
CMap: Connective maps
DILI: Drug-induced liver injury
FDA: Federal drug administration
MCC: Matthews correlation coefficient
MDFS: MultiDimensional feature selection
RF: Random forest
SMILES: Simplified molecular-input line-entry system
