# Supplementary material for: Integration of human cell lines gene expression and chemical properties of drugs for Drug Induced Liver Injury prediction
Source: Biol Direct. 2021 Jan 9;16:2. doi: 10.1186/s13062-020-00286-z (PMC7796564; doi:10.1186/s13062-020-00286-z)
Supplement: Supplementary file 1 — Additional file 1 Supplementary materials for “Integration of human cell lines gene expression and chemical properties of drugs for Drug Induced Liver Injury prediction” [file 13062_2020_286_MOESM1_ESM.pdf]

Supplementary materials for "Integration of human cell lines gene expression and chemical properties of drugs for Drug Induced Liver Injury prediction"

Lesiński et al.

This section describes the hyperparameters selection process. In most cases, our feature selection methods reported no relevant variables in gene expression data sets. Nevertheless, for some cell lines, the number of very weakly informative variables greatly exceeded the expected values, see Figure 1. For example, for the MCF7 cell line, the expected false discovery rate for 100 most relevant variables was near 0.5, suggesting that there are about 50 truly, albeit weakly, informative variables within the 100 most relevant ones, see Figure 2. On the other hand, the measured relevance of variables on the PC3 cell line conformed to the theoretical distribution, and expected false discovery rate is close to 1, see Figures 3 and 4. Therefore, all models were built using top N highly ranked descriptors, with the value of N established experimentally, see Table 1. In the case of molecular descriptors obtained from the Mordred, the number of relevant variables obtained for the entire data set is 127 when FDR level 0.1 was applied, see Figures 5 and 6. The number of relevant variables obtained in cross-validation varies between folds. We used top 100 variables for consistency between folds and also with gene expression data.

The value of the $m$try parameter of Random Forest, corresponding to the number of variables tested at each split creation, was established experimentally outside of the cross-validation loop. All values from the (2,20) interval were tested and AUC was used as a quality metric, see Table 2. The quality of the results was generally not dependent on the selection of $m$try, except when very small values were used. Therefore, the default value of $m$try was used throughout the study.

Table 1: Results (AUC) of feature selection analysis. Number of top ranked variables used in modelling is in the first column.

Var number	MCF7	A549	VCAP
5	0.57	0.51	0.51
10	0.58	0.54	0.52
15	0.58	0.54	0.53
20	0.58	0.55	0.54
25	0.59	0.55	0.54
30	0.59	0.55	0.55
35	0.59	0.55	0.55
40	0.59	0.55	0.55
45	0.60	0.55	0.56
50	0.59	0.56	0.56
60	0.60	0.55	0.56
70	0.61	0.55	0.57
80	0.61	0.56	0.57
90	0.62	0.57	0.57
100	0.62	0.57	0.58
125	0.62	0.57	0.58
150	0.62	0.55	0.57
200	0.62	0.55	0.56
300	0.61	0.55	0.56
400	0.62	0.54	0.55
500	0.61	0.54	0.55

Table 2: Selection of $m$try Random Forest hyper-parameter. Mtry value is in the first column, AUC of models built on to 100 high ranked variables in the next.

$m$try value	MCF7	A549	VCAP
2	0.61	0.54	0.55
3	0.61	0.55	0.56
4	0.62	0.56	0.56
5	0.62	0.56	0.57
6	0.62	0.57	0.57
7	0.62	0.57	0.57
8	0.62	0.57	0.58
9	0.62	0.57	0.58
10	0.62	0.57	0.58
11	0.62	0.57	0.58
12	0.62	0.57	0.58
13	0.62	0.57	0.57
14	0.62	0.57	0.58
15	0.62	0.57	0.58
16	0.62	0.56	0.58
17	0.62	0.57	0.58
18	0.62	0.57	0.58
19	0.62	0.57	0.58
20	0.62	0.56	0.58

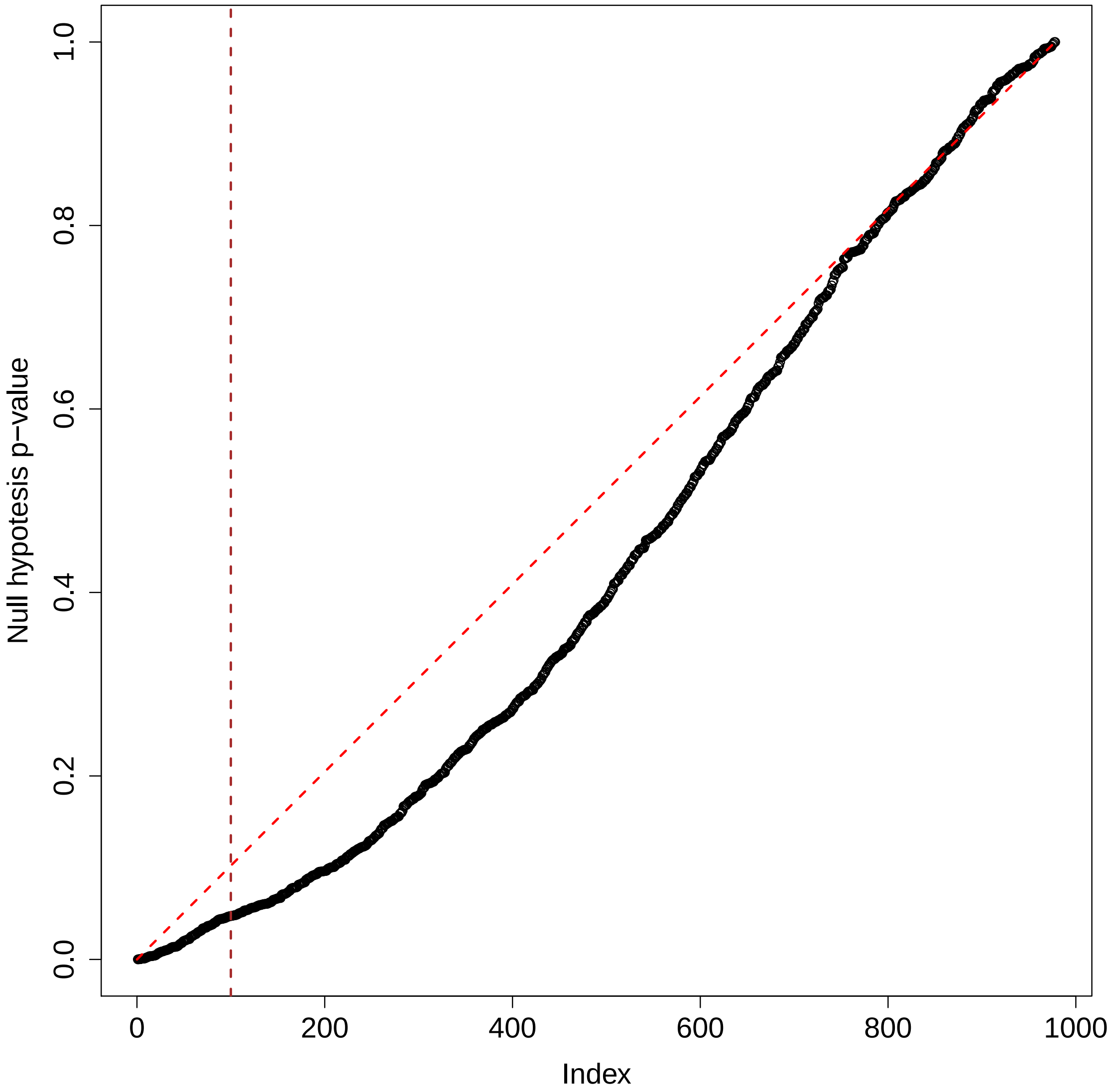

Figure 1: P-value of variables from MCF7 cell line

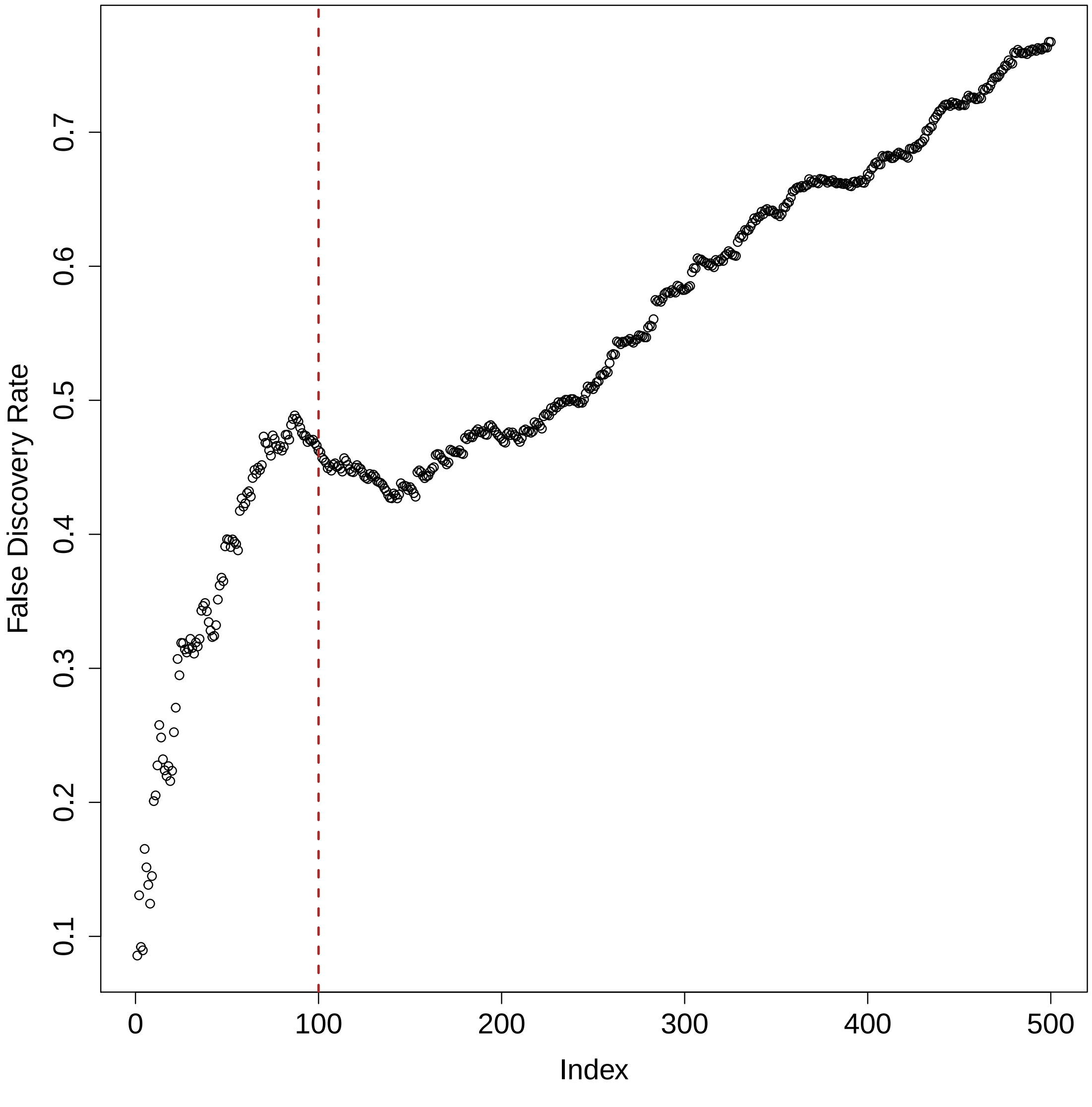

Figure 2: False discovery rate of variables from MCF7 cell line

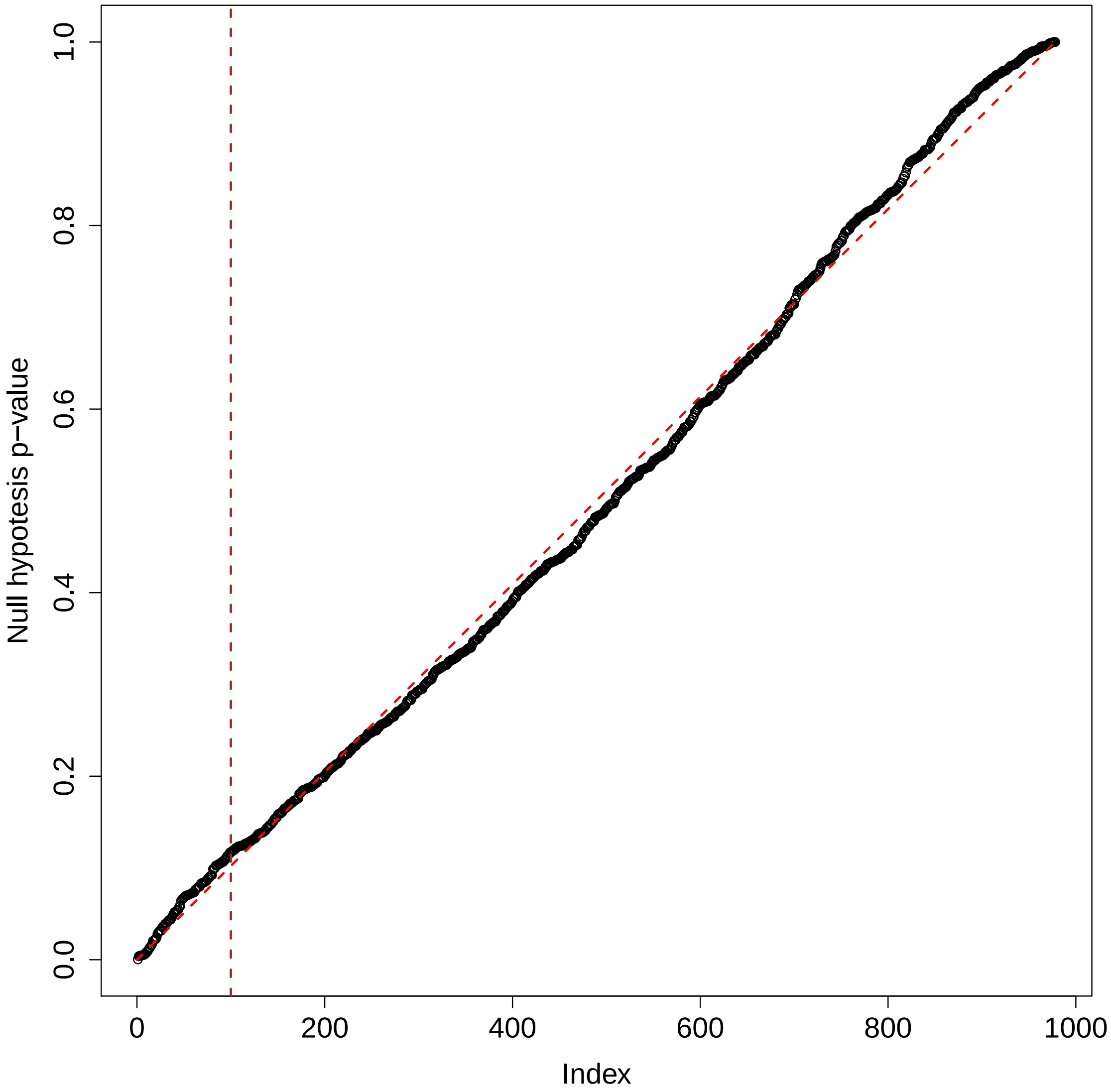

Figure 3: P-value of variables from PC3 cell line

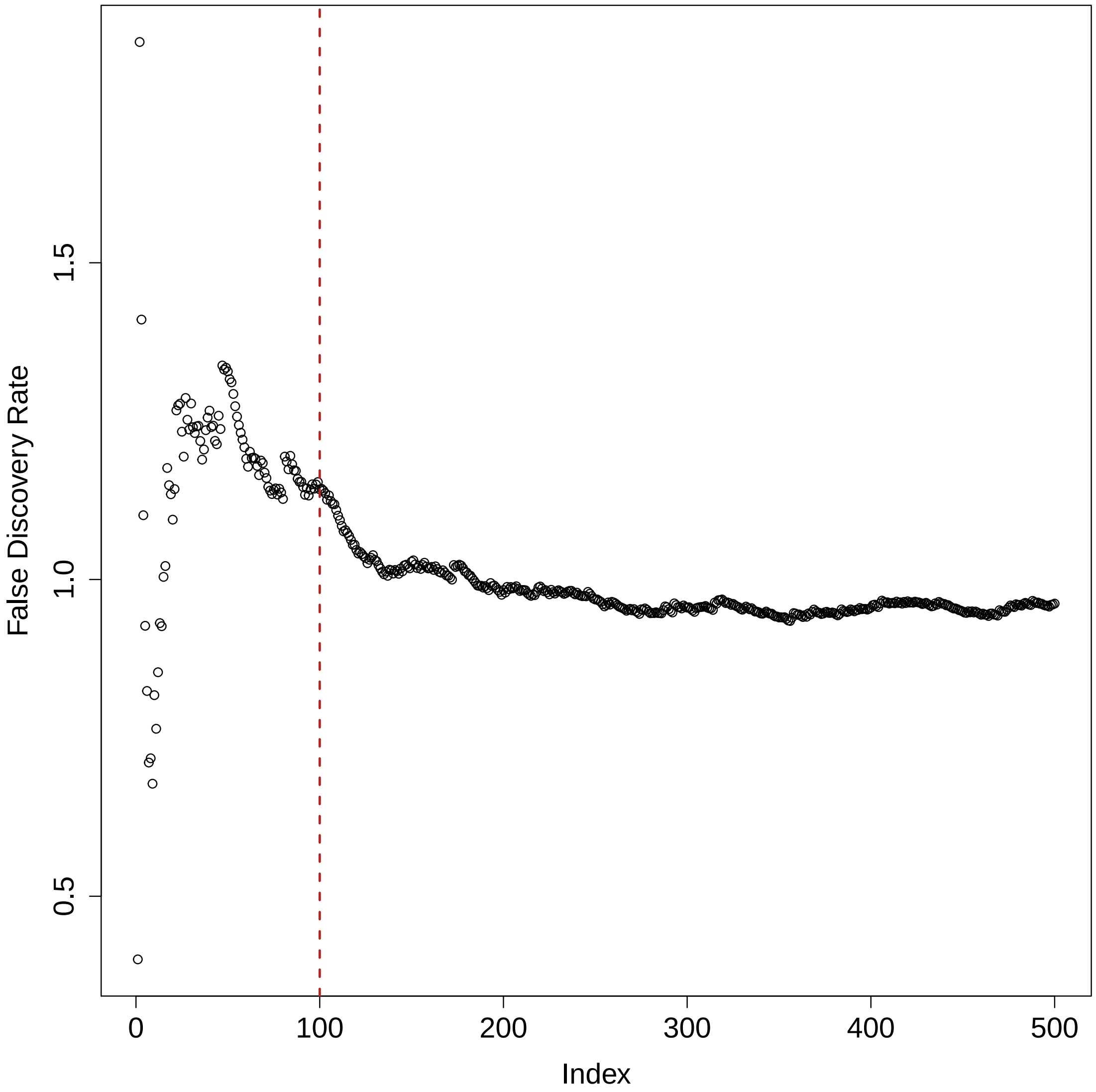

Figure 4: False discovery rate of variables from PC3 cell line

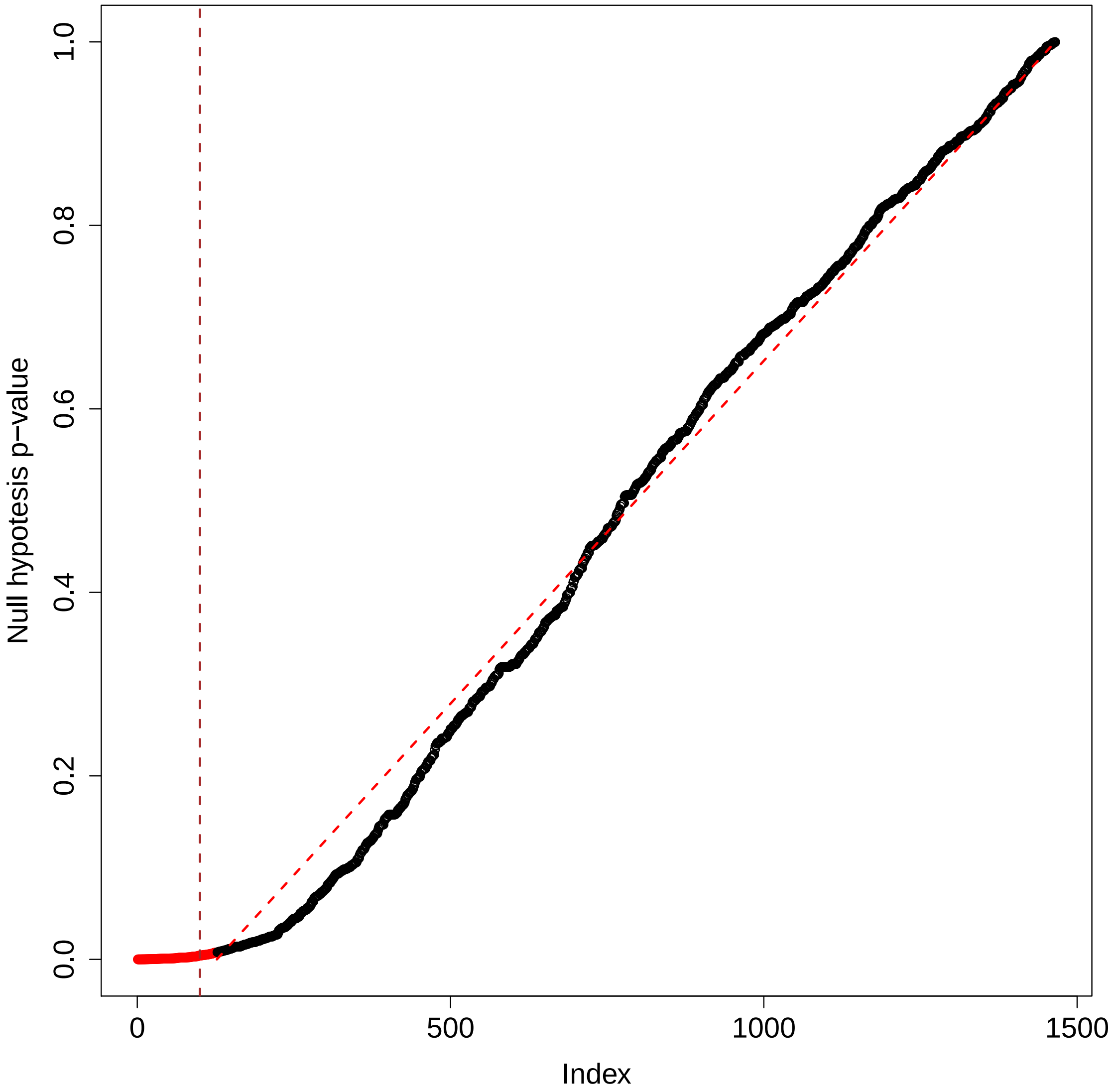

Figure 5: P-value of variables from chemical properties of drugs

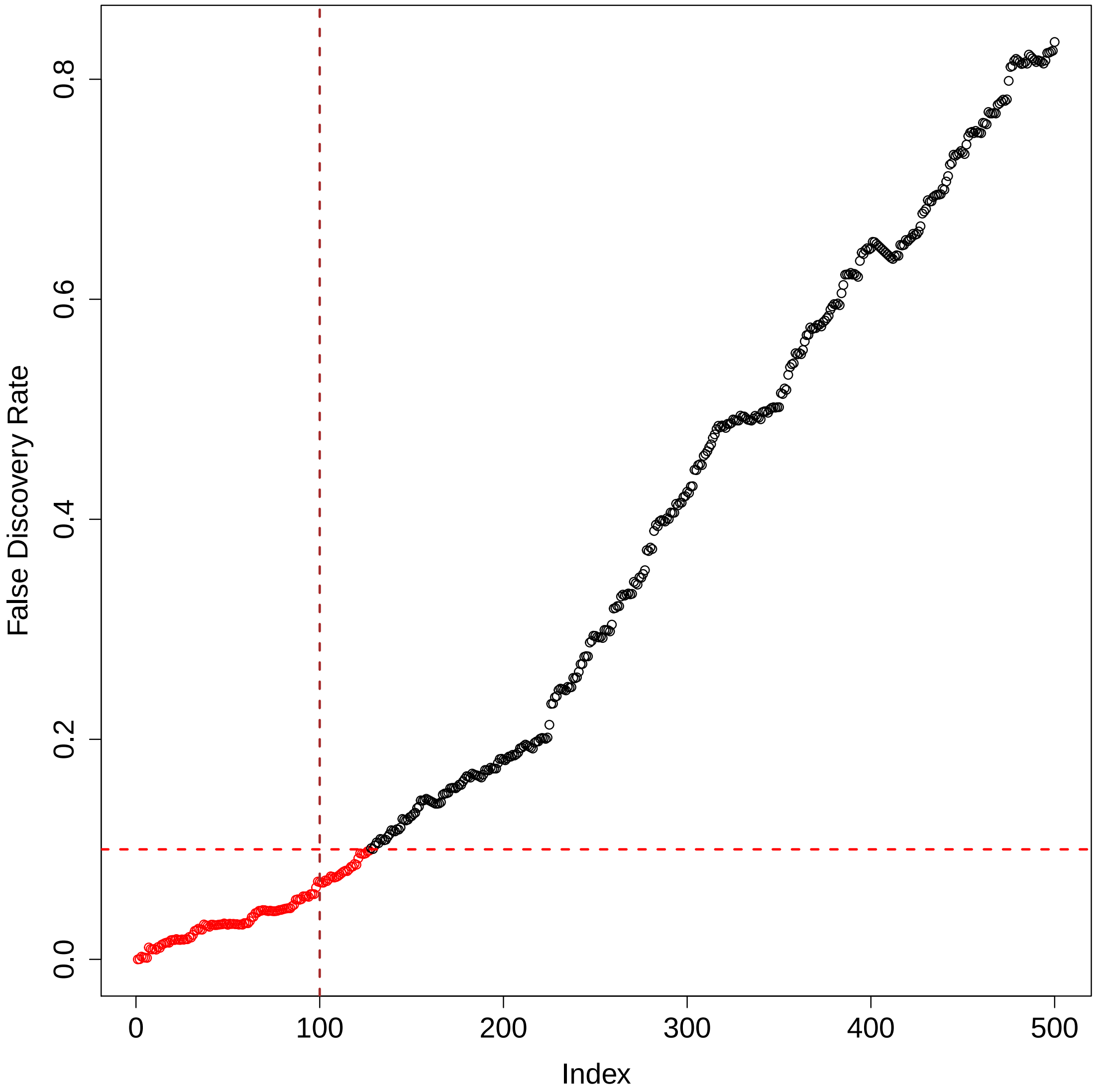

Figure 6: False discovery rate of variables from chemical properties of drugs
